# Prototype of a computerized scale for the active search for potential organ donors ^1^

**DOI:** 10.1590/1518-8345.1936.2930

**Published:** 2017-09-12

**Authors:** Érika Fernanda dos Santos Bezerra Ludwig, Marta Cristiane Alves Pereira, Yolanda Dora Évora Martinez, Karina Dal Sasso Mendes, Mariana Angela Rossaneis

**Affiliations:** 2 MSc, Assistant Professor, Departamento de Enfermagem, Centro Universitário Filadélfia, Londrina, PR, Brazil.; 3 PhD, Professor, Escola de Enfermagem de Ribeirão Preto, Universidade de São Paulo, PAHO/WHO Collaborating Centre for Nursing Research Development, Ribeirão Preto, SP, Brazil.; 4 PhD, Full Professor, Escola de Enfermagem de Ribeirão Preto, Universidade de São Paulo, PAHO/WHO Collaborating Centre for Nursing Research Development, Ribeirão Preto, SP, Brazil.; 5 PhD, RN, Escola de Enfermagem de Ribeirão Preto, Universidade de São Paulo, PAHO/WHO Collaborating Centre for Nursing Research Development, Ribeirão Preto, SP, Brazil.; 6 PhD, Professor, Departamento de Enfermagem, Universidade Estadual de Londrina, Londrina, PR, Brazil.

**Keywords:** Brain Death, Tissue and Organ Procurement, Nursing, Hospital Information Systems, Medical Records Systems, Computerized

## Abstract

**Objective:**

to develop a prototype of a computerized scale for the active search for potential organ and tissue donors.

**Method:**

methodological study, with the analysis of 377 electronic medical records of patients who died due to encephalic death or cardiorespiratory arrest in the intensive care units of a tertiary hospital. Among the deaths due to cardiorespiratory arrest, the study aimed to identify factors indicating underreported encephalic death cases. The Acute Physiology and Chronic Health Evaluation II and Sepsis Related Organ Failure Assessment severity indexes were applied in the protocols. Based on this, a scale was built and sent to five experts for assessment of the scale content, and subsequently, it was computerized by using a prototyping model.

**Results:**

34 underreported encephalic death cases were identified in the medical records of patients with cardiorespiratory arrest. Statistically significant differences were found in the Wilcoxon test between the scores of hospital admissions in the intensive care unit and the opening of the encephalic death protocol for both severity indexes.

**Conclusion:**

the prototype was effective for identifying potential organ donors, as well as for the identification of the degree of organ dysfunction in patients with encephalic death.

## Introduction

The organ donation and transplantation process consists of actions that make it possible to transform Potential Donors (PD), patients with Encephalic Death (ED) protocols, into effective organ and/or tissue donors, aiming at transplantation^1^. The clinical criteria for ED are set out in the Resolution 1480/1997 of the Federal Council of Medicine and, in order to identify patients who meet these criteria, a daily intensive search should be carried out in Intensive Care Units (ICUs) and emergency units^2^ ^-^ ^3^.

Despite the need for active search and mandatory reporting of PD, underreporting of potential donors is among the main causes of non-effectiveness of the donation and transplantation of organs and tissues from a deceased donor. Added to this there is the inadequate structure of the hospitals for the diagnosis of ED and care/maintenance provided to PD, as well as the refusal of the relatives to authorize the donation^4^ ^-^ ^5^.

According to the Brazilian Transplant Registry (BTR), in 2016 there was a slight increase in the actual organs donor notification rates^6^. In 2015^7^, there were 47.8 organ donor notifications per million population (pmp), which corresponds to 9,698 PD, but only 2,854 (14.1 pmp) became effective donors. From January to September 2016, there were 50 notifications and 14.4 donors pmp; however, this increase, from 14.1 to 14.4 pmp, was lower than the expected 15.0 pmp, to reach the goal of 16 donors pmp, by the end of 2016^6^. There are still 34,147 people waiting for organ transplants^7^.

In order to increase the number of transplants from a deceased donor, it is necessary not only to identify the PD, but also to perform it early because ED process culminates in pathophysiological changes resulting from the inactivation of pressure, hormonal and respiratory control centers, which can cause the circulatory collapse and make the donation and transplant process unfeasible^8^.

In this scenario, it is desirable to implement information systems that ensure an active search for the notification and maintenance of PD in the health services throughout the country, using computerized resources capable of identifying data in electronic medical records of critical patients who meet the established criteria for organ and tissue donation. The objective of this study was to develop a prototype of a computerized scale for the active search for potential organ and tissue donors.

## Method

It is a methodological study. The study was carried out in a high complexity hospital, a reference in renal and cardiac transplants, located in the South of Brazil.

The study consisted of 377 electronic medical records of patients who died in 2014 in the Adult Intensive Care Units (A-ICU), aged between 18 and 80 years (age limit for solid organ donation). Data collection was carried out from September to October 2015.

The variables collected from all medical records were: age, sex, diagnosis and length of hospitalization. The medical records of patients with Cardiopulmonary Arrest (CPA) were analyzed in terms of clinical evolution in order to identify encephalic death, brain death, coma with areflexia and/or description of clinical signs of encephalic death, in accordance with current legislation^2^ for identification of underreported ED cases. Medical records that had at least three clinical signs of ED and for which there was no search for potential organ donors were considered as underreported ED cases.

The *Sepsis Related Organ Failure Assessment* (SOFA) and *Acute Physiology and Chronic Health Evaluation* II (APACHE II) prognostic indexes were applied in the medical records of patients with ED. These prognostic indexes or severity indexes evaluate the clinical and laboratory data of the patients, as well as indicate the degree of organic dysfunction, expressed as a numerical value^9^. The APACHE II calculation is obtained through the sum of three items: age, physiological variables (temperature, mean arterial pressure, heart rate, respiratory rate, oxygenation, arterial pH, sodium, potassium, creatinine, hematocrit and leukocytes) and chronic disease diagnosis^10^. The SOFA index is estimated by means of a scoring system, ranging from 1 to 4 points, based on the evaluation of six organic systems: respiratory, hepatic, cardiovascular, renal, central nervous system and coagulation^9^. SOFA and APACHE II scores were calculated on ICU admission, opening of ED protocol, proper ED diagnosis, and on the day of occurrence of CPA, if the latter has occurred before the conclusion of the protocol.

After identifying the feasibility of obtaining these data from patients’ medical records, the first version of the computerized scale for the active search for potential organ donors was build. The procedure for assessment of the scale content consisted of a careful analysis of its dimensions. For this purpose, a panel of judges was created, which was composed of two nurses and three physicians with professional experience of at least two years in the teaching or clinical practice of organ donation and transplant. These profesionals were active in the Center of Notification, Procurement and Distribution of Organs (CNCDO), Organ Procurement Organization (OPO) or Intra-Hospital Committee for Donation of Organs and Tissues for Transplant (CIHDOTT). The selection of these professionals was done through a search in the Lattes Curriculum and they were invited to participate via email. The instrument and the instructions for the assessment process were sent to the evaluators after confirming their acceptance to participate. A period of 30 days was set for the return of the material via email.

The experts evaluated each item of the scale and chose between two options: agree (if you do not want to make changes in the item) and disagree (if you want to propose any change in the item). The judges were also asked to make comments and give suggestions for the improvement of the items, if they considered necessary.

Subsequently, a second version of the scale was generated, which was computerized, by means of the prototyping model, according to Pressman’s theoretical framework (2011)^11^. The prototyping technique began with the communication between the customer and the computer service, to define the general objectives of the prototype. After the presentation of the initial prototype, assessment sessions were conducted between the researcher and the information technology team, until the stabilization of the prototype and creation of the final version. The Delphi tool was used for computerizing the scale, so that data already in the patient’s electronic medical record, such as personal data were automatically entered by the “auto completing” function when the patient’s service number was entered. Subsequently, all further data were entered and registered in the computerized database of the institution.

Data were tabulated and analyzed using the *Statistical Package for the Social Sciences* (SPSS), version 21.0. The Wilcoxon test was applied to compare the APACHE II and SOFA scores, with a significance level α=0.05.

The Research Ethics Committee of the Nursing School of Ribeirão Preto, USP, approved this study in accordance with the Opinion number 119/2015.

## Results

In total, 377 electronic medical records of patients who died in the ICU of the institution under study were analyzed to identify the feasibility of obtaining data that enabled an active search for ED cases, as well as underreporting of PD who have had CPA and showed clinical signs of ED.

It was found that 346 (91.8%) deaths occurred due to CPA and 31 (8.2%) due to ED. In the characterization of the deaths due to CPA, it was evidenced that the majority were male (58.7%); aged equal to or greater than 65 years (49.5%), with a mean age of 60.6 years (Standard Deviation-SD=14.1); predominant length of hospital stay between 8 and 14 days (24.3%), with an average of 23.6 days (SD=95.5).

There were 34 underreported ED cases among the deaths due to CPA, and of these, seven (20.6%) had signs of ED registered in their daily medical clinical evaluations. In the other medical records, 27 (79.4%), signs of ED were described in the daily clinical evaluations performed by doctors and nurses, namely: apperceptive coma, fixed and dilated pupils, absence of corneal reflex and absence of cough reflex. It is noteworthy that, of the 34 medical records, five (14.7%) reported absence of brainstem signs in the clinical evaluations, however, the continuous infusion of sedation to the patient remained. Based on this information, it was possible to include a scale for the evaluation of sedated patients in the first version of the prototype, and the Ramsay scale was chosen for this purpose.

Regarding the characteristics of the patients with ED, there was a predominance of males (21; 67.7%), with a mean age of 48 years (SD=14.5), predominant length of hospital stay of 4 to 7 days (42%), with an average of 8.7 (SD=6.4) days. Only 15 patients (48.4%) completed the ED protocol.

The results of cerebral angiography, a supplementary examination required by the institution in which the study was carried out, were also analyzed for the determination of ED. 

**Figura 1:**
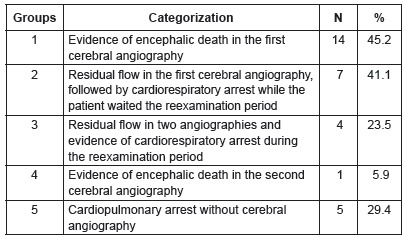
Categorização dos pacientes em protocolo de morte encefálica, segundo realização de exame complementar, constatação de morte encefálica ou evolução para parada cardiorrespiratória (N=31). Ribeirão Preto, SP, 2015

It was observed that of the 31 patients, only 14 (45.2%) were diagnosed with ED in the first cerebral angiography. Thus, these patients were categorized according to the cerebral angiography and determination of encephalic death, or CPA before the determination of encephalic death (Figure 1).

Thus, APACHE II and SOFA scores were estimated for all patients with ED protocol at ICU admission, opening and outcome of the protocol, according to the categorization groups.

In the comparison, using the Wilcoxon test, statistically significant differences were found only between the ICU admission scores and opening of the ED protocol for both severity indexes, with p=0.010 for APACHE II and p<0.001 for SOFA. No statistical test was performed for groups 3, 4 and 5, because the number of participants in these groups was very small. However, it was possible to compare the mean values between APACHE and SOFA scores. In relation to group 3, there was an increase of nin points in the average when comparing the scores of the opening of ED protocol and the day of occurrence of CPA, after the second angiography, for APACHE II. The difference in the average SOFA scores for the same group was five points.

For group 5, composed of patients with CPA, and who did not undergo cerebral angiography, a higher average score was observed in comparison to the other groups, in both indexes, at the opening of the protocol. There was an increase of about three points in the average APACHE II scores at the opening of the DE protocol compared to the day of the occurrence of CPA, and the increase was even greater for SOFA, approximately nine points.

Based on these results, the first version of the scale for the active search for PD was developed, considering patient identification data (item 1) and hospital information (item 2), as well as the information necessary to estimate APACHE II and SOFA scores. The Ramsay scale was used for patient evaluation (item 3) with respect to sedation, while the Glasgow Coma Scale (GCS) was used in the evaluation of non-sedated patients. In addition, the neurological examination (item 4), indicative of DE, was considered, since it is already part of the routine of the ICU professionals of the study institution and, finally, APACHE II (item 5) and SOFA (Item 6) severity indexes were also considered.

**Figure 2:**
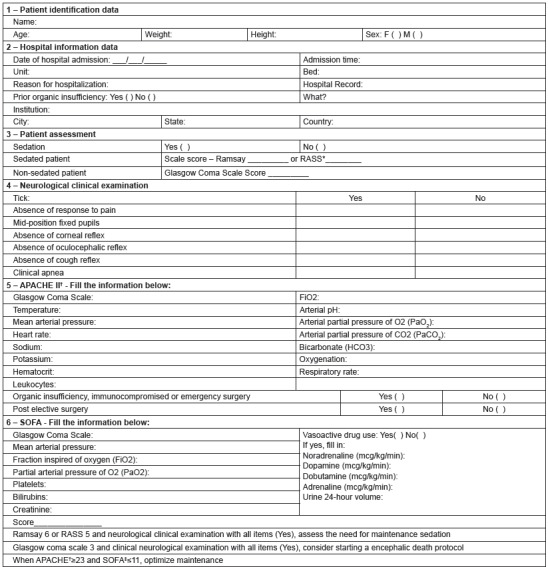
Scale for the active search for potential organ donors, based on APACHE II and SOFA - following an assessment by the experts. Ribeirão Preto, SP, 2015

Subsequently, the first version of the scale was sent to five professionals for assessment of the scale content. However, one of the professionals did not return the results of the assessment within the requested deadline and was excluded from the study. The changes suggested by the experts were: adding the marital status, name of the parents and spouse and removing the date of birth, since this information is obtained from age, in item 1; adding the name of the institution, city, state and country, in item 2; adding, along with the Ramsay scale, the Richmond *Agitation Sedation Scale* (RASS) and replacing the GCS by the Jouvet Coma Scale (JCS), in item 3; adding, at the end of the instrument, the classification of the scores that inform the professional regarding the identification and maintenance of PD in items 5 and 6 (Figure 2).

*Richmond Agitation Sedation Scale; †*Acute Physiology and Chronic Health Evaluation II*; ‡*Sepsis Related Organ Failure Assessment*.

**Figure 3:**
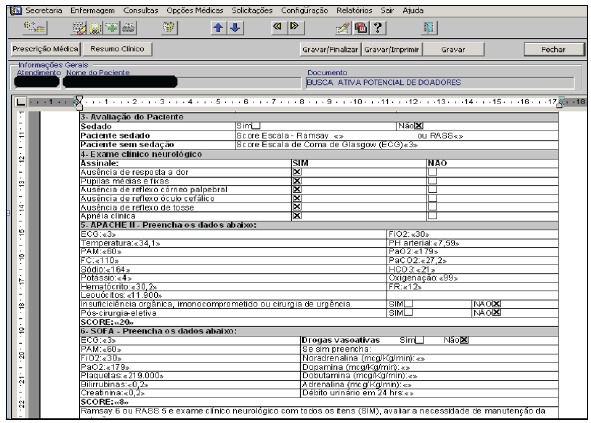
Display of the scale for the active search for potential organ donors inserted into the Electronic Patient Record (EPR). Ribeirão Preto, SP, 2015

Source: Electronic Patient Record

Following an assessment by the experts, the computerization process started (third phase), regarding the construction of the prototype in the computer service of the institution. From the return of the assessment by the experts, with the implementation of the scale in the hospital information system (Figure 3), the first application evidenced the need for adjustments in the estimation of APACHE II and SOFA final scores, with subsequent simulation of use, which allowed finding out the application time, ranging from five to 10 minutes.

## Discussion

The development of the prototype of the computerized scale for the active search for potential donors and its subsequent implementation as part of the patient’s electronic medical record, allowed rapid access to information, agility in use and sharing of data among health professionals, as inherent advantages of the Electronic Patient Record (EPR). Its assessment by the experts allowed the improvement of its content, in addition to conferring a greater credibility to the information for an active search for all potential donors.

It should be noted that the suggestion to include the available data into EPR (marital status, mother’s name, father’s name and spouse’s name) was not accepted, since such information does not correspond to the main purpose of the daily active search and leads to an increase in application time.

Regarding item 3, it was requested to include the RASS scale, so the health service could choose between using the Ramsay scale or RASS, if the active search scale was used in another health institution, since both scales are widely used in ICUs. Sedation is a fairly common therapeutic method, including at ICUs, and its practice should be re-evaluated regularly through the systematic use of a validated scale^12^.

The Ramsay scale assesses the degree of sedation of patients, with variations in the scale from 0 to 6 for post-stimulus patient responses, ranging from grade 1 to anxious and agitated patients to grade 6, to asleep and unresponsive patients^13^. The RASS scale, with scores ranging from +4 (combative) to -5 (coma, does not wake up), indicates five stages of sedation, with negative values and five agitation stages, with positive values^14^ ^-^ ^15^.

Thus, patients with a score 6 on the Ramsay scale, or -5 on the RASS scale, associated with the presence of clinical signs of ED, which correspond to item 4 of the scale for the search active for PD, must be evaluated regarding the indication of maintenance of sedation. If sedation is discontinued and the patient maintains arreflexia and GCS with a value equal to 3, the opening of an ED protocol must be initiated^14^.

Also regarding item 3, one of the experts suggested replacing GCS by the Jouvet Coma Scale (JCS), using as justification the fact that GCS evaluates trauma, while JCS evaluates specific reflexes of brainstem, as well as pupillary reflexes. The replacement was not performed on the active search scale, as both coma scales were used to assess the level of consciousness, that is, the degree of behavioral alertness of the individual^16^.

The JCS was developed to assess the level of consciousness of patients in a persistent vegetative state. However, studies have shown its applicability in patients in an acute vegetative state. It is characterized by being highly sensitive and evaluating variations in the level of consciousness close to the normal state, since it evaluates the cortical and brainstem functions, however, it is a scale of difficult application, and for this reason, the health professional must be qualified to apply it^16^.

GCS is the most widely used scoring system at the international level for evaluating comatose patients under intensive care, with the aim of standardizing clinical observations in adults with neurological damage associated with alterations of consciousness. Its score ranges from 3 to 15, and score 3 is compatible with brain death, when associated to the evaluation of other parameters^14^ ^-^ ^16^.

The application of Ramsay or RASS scales for sedated patients, and GCS for non-sedated patients, associated with the evaluation of clinical signs of ED, as elements that make up the scale for the active search for potential organ donors, become indispensable tools in the identification of these patients. The neurological evaluation and the scales scores will help health professionals in deciding on the opening of an ED protocol. The objective of this study corroborates the evidence demonstrated by the results that the neurological clinical examination, indicative of ED, is part of the routine of the intensive care professionals of the study institution, as well as the Ramsay scale and GCS. Therefore, the development of a computerized scale, containing the clinical neurological examination, will not burden the professional with one more task, but will help in the identification and notification of all PD, making possible the organ and tissue donation process aiming at transplantation.

According to the Brazilian Association of Organ Transplantation^6^ (ABTO), underreporting decreased in Brazil: in 2004, there were 40.75 underreported cases pmp; in 2012, there were 27.9 cases pmp; and in 2013, 23.5 cases pmp. However, underreporting should be abolished.

In Brazil, in 2007, there were 4,714 notifications and, in 2014, 9,351 PD were notified. However, the Brazilian goal is to reach 20 donors pmp in 2018, with a notification rate of 55 PD pmp in 2018. In 2014, these 9,351 notifications corresponded to 49 PD pmp, and in 2015, there was a decrease in the number of PD notifications (47.8 pmp), confirming the importance of identifying and notifying all PD^6^. Underreporting corroborates the limited supply of organs and restricts transplants, a limitation that affects not only Brazil. According to the International Registry of Organ Donation and Transplantation (IRODaT) (2010), organ shortage is one of the main challenges for the proper management of organ donation and transplantation worldwide^17^.

The search for instruments for the identification of indicators capable of helping in the development of strategies to improve the organ donation and transplantation process and to increase the number of effective donors, with a consequent increase in the number of transplants, is a global need, since organ shortage is a problem affecting several countries. A fact that encourages countries to develop audits in their ICUs, seeking to know the number of EDs, the number of underreported PD cases and the causes of PD losses^18^.

In the organ donation and transplantation scenario, Spain occupies a privileged position, with the highest rates ever registered, maintaining values between 33 and 35 donors pmp in recent years. However, in the 1980s, this rate was 14 donors pmp. The significant increase in the donation rate was the result of the implementation of several measures, mainly those related to organization, together with the internationally known Spanish model for Organ Donation and Transplantation, which was based on two basic principles: organization and continuos adaptation to the new knowledge produced in this area^19^.

Spain is one of the countries that have stood out for the use of quality instruments capable of defining the theoretical capacity of organ donation, according to the type of hospital, of detecting possible losses of donors during the donation process, and of analyzing the causes of these losses, identifying which hospital factors have an impact on the donation process. In Brazil, effective instruments to measure and evaluate the donation and transplantation process are needed^18^.

In this way, the scale for the active search for potential organ donors, based on APACHE II and SOFA severity indexes, will serve as an instrument for organizing the organ donation and transplantation process in the institution of the study. In addition to the identification and notification of all patients with ED, this scale will allow the maintenance of PD, reducing avoidable losses, increasing the quantity and quality of organs available for transplantation, and providing effectiveness in the organ donation and transplantation process.

Avoidable losses are characterized by failures in the maintenance of PD, leading to CPA. Although the actions necessary for the adequate maintenance of PD seem obvious, their accomplishment does not occur in most ICUs, which is evidenced by the almost absolute absence of systematization in the care provided to the PD of multiple organs^20^.

In the results of this study, it was observed that several patients presented CPA without determination of ED, which prevents the organ allocation process. In addition, the values of the APACHE II and SOFA severity indexes showed a statistically significant difference between the scores at ICU admission and at the opening of the ED protocol, by means of the application of the Wilcoxon test. This difference not only represents the worsening of the condition, but also a physiological dysfunction that can cause CPA in the PD.

Severity and organ dysfunction scoring systems, such as APACHE II and SOFA, have been widely used and validated in various settings and populations^21^. Many of the physiological changes inherent to ED are considered in these indexes and can guide the maintenance performed by the health professional, in addition to that, SOFA allows the individual evaluation of each organ. The APACHE II and SOFA average scores achieved in the first cerebral angiography for the group of patients with ED, defined the values described in the caption of the scale for the active search for PD of organs, with the objective of guiding the professionals in the optimization of PD maintenance.

Thus, APACHE II and SOFA severity indexes, which make up the computerized active search scale, proved to be effective in the maintenance of PD, and corroborated the importance of daily application of this instrument in ICUs. It should be noted that, in this study, these indexes proved to be effective in guiding the maintenance of PD, but not in the identification of PD. This task was assigned to items 3 and 4 of the scale for the active search for potential donors.

It is highlighted as an additional potentiality of this study, the possibility of applying the active search scale also retrospectively in the medical records, with the objective of identifying the number of PD, as well as its effectiveness in the determination of ED.

The calculation of ED rate in a health institution is one of the main obstacles to the detection of PD worldwide. In Brazil, estimation of potential donors is calculated according to the population rate of a given area, to the different hospital indexes, and to the percentages of deaths in ICUs^18^.

The estimated incidence of ED is 60 cases per million inhabitants per year, which corresponds to 12% of the deaths occurred in intensive care units of large general hospitals^22^. Adding the number of underreported cases, 34, to the number of patients with ED, 33, 67 notifications are obtained, ie, a result that far exceeded the 12% estimate, reaching 14.4%. This may be the specific estimate of potential donors at the study institution.

It is worth mentioning that, importing the data registered in the EPR may be a limiting factor for the effectiveness of the active search scale in terms of identification of PD due to the repetition of the information provided by EPR. This may be due to the possible existence of unreliable data for the clinical neurological examination or unreliable scores obtained in APACHE II and SOFA. In addition, the scale has not been submitted to the validation process, a future stage that will be developed for the continuation of this study.

## Conclusion

The prototype was efficient to identify potential donors and effective organ donors, as well as to help in the maintenance of these individuals, by means of APACHE II and SOFA severity indexes, with a view to the accomplishment of a possible transplantation. In addition, it made possible the identification of patients in whom the diagnosis of ED was underreported by the multiprofessional team.

It is expected that this scale can be used in other institutions, with a view to improving the identification and maintenance of potential donors, with an impact on the reduction of losses due to underreporting cases and inadequate maintenance of potential donors, thus allowing a greater effectiveness in the supply of organs for transplantation.
